# Specific Tumor Localization of Immunogenic Lipid-Coated Mesoporous Silica Nanoparticles following Intraperitoneal Administration in a Mouse Model of Serous Epithelial Ovarian Cancer

**DOI:** 10.3390/cancers15184626

**Published:** 2023-09-19

**Authors:** Achraf Noureddine, Benjamin Marwedel, Lien Tang, Lorel Y. Medina, Rita E. Serda

**Affiliations:** 1 Department of Chemical and Biological Engineering, University of New Mexico, Albuquerque, NM 87131, USA; anoureddine@unm.edu (A.N.); lienttang@unm.edu (L.T.); 2 Department of Internal Medicine, University of New Mexico Health Science Center, Albuquerque, NM 87131, USA; bmarwedel@salud.unm.edu (B.M.); lymedina@salud.unm.edu (L.Y.M.)

**Keywords:** mesoporous silica, liposome, MPL-A, ovarian cancer, intraperitoneal delivery, major histocompatibility complex, endosome streaming

## Abstract

**Simple Summary:**

Ovarian tumors mobilize a heterogenous population of myeloid cells. While these cells initially contribute to anti-tumor immunity, as cancer progresses, they switch from immunostimulatory to immunosuppressive. Ovarian cancer is most frequently restricted to the peritoneal cavity, negating the need for systemic delivery of therapeutics. With a goal of targeting and activating myeloid cells in the tumor microenvironment, this study sought to determine which nanoparticle characteristics enhance uptake by myeloid cells in vitro and in vivo using an animal model of ovarian cancer. Lipids coating a mesoporous silica core were optimized for surface charge, formulation, and inclusion of immunogenic monophosphoryl lipid A. Benefits of a mesoporous core include opportunities for loading with additional therapeutics, such as antigens to direct immune responses or other therapeutic payloads that facilitate anti-cancer immune responses.

**Abstract:**

Immunogenic lipid-coated mesoporous silica nanoparticles (ILM) present pathogen-associated molecular patterns (PAMPs) on the nanoparticle surface to engage pathogen-associated receptors on immune cells. The mesoporous core is capable of loading additional immunogens, antigens or drugs. In this study, the impact of lipid composition, surface potential and intercalation of lipophilic monophosphoryl lipid A (MPL-A) in the lipid coat on nanoparticle properties and cellular interactions is presented. Loading and retention of the model antigen ovalbumin into the mesoporous silica core were found to be similar for all nanoparticle formulations, with presentation of ova peptide (SIINFEKL) by major histocompatibility complex (MHC) evaluated to facilitate the selection of an anionic nanoparticle composition. ILM were able to induce lysosomal tubulation and streaming of lysosomes towards the cell surface in dendritic cells, leading to an enhanced surface presentation of MHC. Myeloid cells robustly internalized all ILM formulations; however, non-myeloid cells selectively internalized cationic ILM in vitro in the presence of 20% serum. Interestingly, ILM administration to the peritoneal cavity of mice with disseminated ovarian cancer resulted in selective accumulation of ILM in tumor-associated tissues (>80%), regardless of nanoparticle surface charge or the presence of MPL-A. Immunofluorescence analysis of the omental tumor showed that ILMs, regardless of surface charge, were localized within clusters of CD11b^+^ myeloid cells 24 h post administration. Selective uptake of ILMs by myeloid cells in vivo indicates that these cells outcompete other cell populations in the ovarian tumor microenvironment, making them a strong target for therapeutic interventions.

## 1. Introduction

Acute bacterial infection has been associated with spontaneous cancer regression through the non-specific activation of a suppressed immune system [1]. The innate immune system recognizes bacterial pathogens or their associated molecules through expression of Toll-like receptors (TLRs) [2]. TLR4, a receptor for Gram-negative bacterial lipopolysaccharide (LPS), is expressed by dendritic cells (DC), tumor-associated macrophages (TAMs), cytotoxic T cells, myeloid-derived suppressor cells (MDSC), and some stromal cells [3]. The presence of TLR4 on the plasma membrane of immune cells enables prompt recognition and defense against invading pathogens [4]. Pathogen-associated molecular patterns (PAMPs), such as monophosphoryl lipid A (MPL-A), derived from LPS, are commonly used in vaccine formulations [5,6]. Activation of TLR4 on immune cells leads to the dimerization and activation of two intracellular signaling cascades. One cascade, induced within the plasma membrane (MyD88-dependent), leads to rapid induction of pro-inflammatory cytokines, while the second cascade, localized within early endosomes (TIRF-dependent), induces a type I interferon response [7]. Activation of these proinflammatory pathways promotes immune responses, facilitating the formation of immunological memory against pathogen-associated antigens.

Ongoing challenges for the use of nanoparticles as drug delivery carriers for cancer therapy include low tumor accumulation [8]. While active targeting of cancer cells with nanoparticles is an attractive concept, a 2016 mega-analysis by Chan and Colleagues [9] showed that only 0.7% of intravenously delivered nanoparticles reach the tumor. Once injected into the blood, immediate opsonization of nanoparticles with serum proteins creates an outer corona that leads to the rapid uptake by phagocytic cells, clearance from the blood, and a high accumulation in the liver and spleen. In 2018, Chan and colleagues reported that 24 h post intravenous injection, just 0.0014% of gold nanoparticles are associated with cancer cells, with active targeting failing to improve tumor accumulation compared to passive targeting [8]. Myeloid cells, such as macrophages, are among the primary cell types that internalize nanoparticles [10]. In the Chan study, a large proportion of the gold nanoparticles (7–38 times more) in tumors were associated with TAMs located in the perivascular space, despite 58% of the tumor cell population being cancer cells (EpCAM^+^) and only 14% being F4/80^+^ TAM.

Ovarian tumors mobilize a heterogenous population of myeloid cells [11]. While these cells initially contribute to anti-tumor immunity, as cancer progresses, they switch from immunostimulatory to immunosuppressive [12]. In an effort to benefit from high nanoparticle uptake by myeloid cells, this study coated nanoparticles with microbial molecules to facilitate targeting and activation of myeloid cells. Lipid-coated mesoporous silica nanoparticles (LM) were chosen to integrate MPL-A into the lipid coat, positioning it for targeting and activation of TLR4 on immune cells (creating immunogenic LM: ILM), and to enable loading of antigens (here we used the model antigen ovalbumin) into the porous core. While in vitro studies demonstrated that both cationic and anionic LM containing MPL (ILM) were able to upregulate the costimulatory molecule CD40 on DC and be internalized by DC and macrophages, only cationic ILM were internalized by endothelial, stromal, and cancer cells. However, in vivo, myeloid cells were the dominant population associating with ILM, regardless of nanoparticle surface charge. All ILM and LM (no MPL) accumulated predominately in tumor-burdened tissues following intraperitoneal delivery.

## 2. Experimental

### 2.1. Materials

Tetraorthosilicate (TEOS), 3-Aminopropyltriethoxysilane (APTES), cetyltrimethylammonium chloride (CTAC, 25%wt in water), triethanolamine, cyclohexane, succinic anhydride, dimethylformamide (DMF), phosphotungstic acid, hydrochloric acid (HCl, 36%), and ammonium nitrate were purchased from Sigma-Aldrich (St. Louis, MO, USA). Unlabeled duck ovalbumin was purchased from MP-Biomedicals. NHS ester-activated DyLight™ (488, 594, 633) or AlexaFluor™ 647 Fluorophores were purchased from ThermoFisher Scientific. Triethoxypropylsuccinic anhydride was purchased from Gelest (Morrisville, PA, USA). Lipids, including cholesterol, 1,2-dipalmitoyl-sn-glycero-3-phospho-choline (DPPC), 1,2-dimyristoyl-sn-glycero-3-phospho-(1′-rac-glycerol) (sodium salt) (DMPG), and 1,2-dioleoyl-3-trimethylammonium-propane (chloride salt) (DOTAP), were purchased from Avanti Polar Lipids (Alabaster, AL, USA). Only Lipid A, monophos-phoryl from Salmonella enterica serotype minnesota Re 595 (Re mutant) (MPL) was purchased from Sigma-Aldrich. The following antibodies were purchased from Thermofisher (Hillsboro, OR, USA): CD11b FITC (M1/70), CD11c FITC or PE (HL3), SIINFEKL/H-2kb APC (eBio25-D1.16), CD40 FITC (1C10), MHC I PE (I-A/I-E), and MHC II I-AD APC (AM8-32.1).

### 2.2. Synthesis of ~7 nm Dendritic Pores Monodisperse MSNs: Bare (MSN) or Carboxylic Acid-Terminated (MSN-COOH) or Primary Amine-Terminated (MSN-NH_2_)

The synthesis of the MSNs was adapted from previously reported methods [1,2]. In a 100 mL round bottom flask, triethanolamine (0.18 g) was added along with CTAC (24 mL, pH = 6) and water (36 mL). The pH of the solution was adjusted to 8.5 with sodium hydroxide, and the mixture was heated to 50 °C and stirred (600 rpm) for 1 h. The stirring rate was adjusted to 350 rpm and a 20 mL solution of TEOS in cyclohexane (10% *v*/*v*) was slowly added to form a biphase system. For functionalized MSN, a solution of triethoxypropylsuccinic anhydride (125 µL, for MSN-COOH) or APTES (110 µL, for MSN-NH_2_) in ethanol (200 µL) was added next day (t = 16 h) in the bottom aqueous phase (containing silica nanoparticles) and kept reacting for 4 h. The upper organic phase was then carefully removed, and the nanoparticles suspension was centrifuged. The isolated pellet is suspended in ethanol and centrifuged. The surfactant removal was achieved by successive washing steps using NH_4_NO_3_ (6 g/L ethanol) and HCl (1% ethanol, twice); each step included 15 min sonication and centrifugation. All centrifugation cycles were carried out at 50K rcf for 20 min at 18 °C. Lastly, the template-free MSN were washed twice in ethanol and stored as a suspension in ethanol. The suspensions were previously shown to be stable for at least two years [13].

### 2.3. Conjugation of Fluorescent Labels on MSN-NH_2_

DyLight™ 488-, 594-, 633-, and AlexaFluor 647-NHS were used to fluorescently label MSNs for in vitro and in vivo experiments. A solution of dye in DMF (1 mg/mL, 250 µL) stored at −20 °C was added to a suspension of MSN-NH_2_ (10 mg, 2.5 mg/mL) and reacted for 18–24 h at room temperature in the dark. The mixture was centrifuged and resuspended in succinic anhydride solution in DMF (100 mg, 25 mg/mL) and reacted for 24 h at room temperature in the dark (in order to convert the MSNs from a positive to a negative charge). Next, the mixture was centrifuged, and the isolated dyed pellet was washed in DMF (once) then in pure ethanol (twice). An aliquot was washed in water twice to confirm the final negative charge of the fluorescently labeled MSN.

### 2.4. Preparation of Immunogenic Liposomes

Individual lipids in chloroform (1, 10 or 25 mg/mL) are stored under an argon atmosphere at −25 °C. Seven different PEG-free lipid formulations were prepared by mixing the corresponding constituting lipids in a glass vial (in a glovebox) with total amounts ranging from 5 to 15 mg. The chloroform was removed from the lipid mixture under reduced pressure (rotator evaporator, 10 min) then kept under reduced pressure overnight in a vacuum pump in order to remove all chloroform residues. The dry lipid film was then hydrated in phosphate-buffered saline (PBS) to create a 5 mg/mL suspension and sonicated for at least 20 min at 45 °C. The liposomal suspensions were used directly after preparation to form IL or ILM.

### 2.5. Ovalbumin Loading Procedure and ILM Assembly

A fresh solution of ovalbumin in distilled water (1 or 5 mg/mL) was prepared before the loading procedure. Then, MSNs (dye-labeled or not), in water (1 mg), were incubated (gentle shaking) in the OVA solution (with 1/1 MSN/OVA *wt* ratio) for 15 min at room temperature (22 °C) in the dark. Afterwards, the OVA-MSN mixture was centrifuged (21K rcf, 10 min, 4 °C), and the isolated pellet was resuspended in water before immunogenic liposomes (5 mg/mL) were added under sonication (20 s). The obtained mixture was then centrifuged (21K rcf, 10 min, 4 °C), and the isolated pellet was suspended in PBS and centrifuged. The pellet was resuspended in PBS at 1 mg/mL before in vitro and/or in vivo experiments. All supernatants were saved for protein loading quantitation by CBQCA. The procedure was the same with any type of MSN or lipid used.

### 2.6. OVA Loading Quantitation Assay

A CBQCA Protein Quantitation Kit was used to quantify the extent of OVA loading within the ILM complex. The ATTO-TAGTM CBQCA reagent (3-(4-carboxybenzoyl)quinoline-2-carboxaldehyde) reacts with the primary amines of OVA upon addition of KCN. The procedure was carried out according to the ThermoFisher Scientific/Molecular Probe’s recommendations, using bovine serum albumin (BSA) to create a standard curve.

### 2.7. Phosphotungstic Acid-Based Negative Staining of ILM for TEM Imaging

A freshly prepared suspension of ILM in PBS (0.1 mg/mL, 5 µL) was added onto a TEM holey carbon copper grid and dried in air briefly. A phosphotungstic acid (PTA) solution (2% in PBS, 5 µL) was added then quickly removed with a Kimtech^®^ kimwipe after 10–15 s. The grid was then washed in water (15 µL) three times and kept under air for drying (30 min). TEM images were acquired on a JEOL 2010 (Tokyo, Japan) instrument equipped with a Gatan Orius digital camera system (Warrendale, PA, USA) using 200 kV voltage.

### 2.8. ILM Physical Characterization

Nitrogen adsorption–desorption isotherm of MSNs was obtained on a Micromeritics ASAP 2020 at 77 K. Samples were degassed at 40 °C for 12 h before measurements. The surface area was calculated following the Brunauer–Emmet–Teller (BET) equation and the pore size was obtained via DFT theory and standard Barrett–Joyner–Halenda (BJH) method from adsorption and desorption branches. Hydrodynamic size and zeta potential data were acquired on a Malvern Zetasizer Nano-ZS equipped with a He–Ne laser (633 nm) and non-invasive backscatter optics using freshly prepared particles. All samples for DLS measurements were suspended in distilled water or ethanol at a 1 mg/mL concentration. Samples were washed three times through centrifugation prior to measurements. Measurements were acquired at 25 °C. DLS measurements for each sample were obtained in triplicate and then the Z-average diameter (by intensity) was used for all reported hydrodynamic size values. The zeta potential for all the samples was measured in distilled water in triplicate according to Smoluchowski theory [14]. All reported values correspond to the average of three independent measurements.

### 2.9. Cell Culture

RAW 264.7 macrophages (ATCC, Manassas, VA, USA), A549 human lung cancer (ATCC), dermal fibroblasts (Lonza, Bend, OR, USA), human microvascular endothelial cells (HMVEC; Lonza) and bone marrow-derived DC were used for in vitro cell association studies. Cells were cultured at 37 °C in 5% CO_2_ in the vendor recommended media (Invitrogen Corporation, Carlsbad, CA, USA) supplemented with 10% fetal bovine serum (FBS) and penicillin-streptomycin (PS) (ThermoFisher Scientific, Grand Island, NY, USA). Briefly, bone marrow cells were obtained from the femurs of C57BLl/6 mice using 26-guage needles to flush the bone with complete media (RPMI containing 10% FBS, PS and 20 ng/mL GM-CSF) and cultured for 8 days prior to the introduction of ILM [15].

### 2.10. Flow Cytometry Analysis of Particle Association with Cells and Surface Markers

Cells were seeded into 12 well plates at 1 × 10^5^ cells per well and allowed to adhere overnight. Cells were then incubated with 25 µg/well fluorescent ILM for cell uptake (2 h in media containing 20% FBS) or activation studies (10% FBS; 24–72 h as indicated). Cells were labeled with the fluorescent antibodies and analyzed using either a BD™ LSRFortessa flow cytometer using FACSDiva™ software version 6.1.1 (BD Biosciences).

### 2.11. Confocal Microscopy Imaging of Nanoparticle Cellular Association

Cell lines or bone marrow derived DC were seeded onto #1.5 glass cover slips in 6-well plates at a density of 1 × 10^5^ cells per well. After 24 h incubation, fluorescent ILM were added to the cells using fresh complete media containing 10–20% FBS (as indicated) at 25 µg/mL. LysoTracker Green DND-26 or Red DND-99 (75 nM; ThermoFisher Scientific) was added to cells in prewarmed culture media during the final 30 min of incubation. Cells were then washed with PBS, fixed with 4% paraformaldehyde in PBS for 15 min with prewarmed solutions, followed by overnight refrigeration, two washes with PBS, and permeabilization with 0.1% Triton-X in PBS for 15 min. Cells were then blocked with 1% BSA for 20 min and then labeled with 5 units/0.5 mL Rhodamine (or Alexa Fluor™ 647) phalloidin and/or 10 µg/mL mouse anti-α-tubulin antibody-Alexa Fluor 647 in 1% BSA for 1 h. After washing with PBS, slides were mounted using Prolong Gold with DAPI. Confocal images were acquired with a 63×/1.4 NA oil objective in sequential scanning mode using a Leica TCS SP8 confocal microscope.

### 2.12. ILM Biodistribution Studies

Female FVB mice (6–8 weeks old) in groups of three were injected intraperitoneally (IP) with 2 × 10^5^ BR5-Akt-Luc BRCA1- deficient serous epithelial ovarian cancer cells in 200 μL PBS. Nineteen days post tumor challenge (Day 19), mice were administered with IP injection of one of three Alexafluor 647-labeled LM or ILM formulations at 200 μg/200 μL PBS/mouse: anionic (DMPG), cationic (DOTAP) LM or DMPG ILM (containing MPL-A). After 24 h, the mice were IP-injected with 150 mg/kg D-luciferin potassium salt for bioluminescent and fluorescent imaging using the Xenogen IVIS^®^ Spectrum (Perkin Elmer, Waltham, MA, USA). Mice were then euthanized, and images were taken of the abdominal region, both open and closed. Peritoneal tissues were harvested and imaged. Regions of interest (ROI) were created around each organ/tissue, and total photon counts per second (p/s) were determined and compared across groups. Tissues were frozen in O.C.T. Compound and stored at −80 °C.

### 2.13. Tissue Immunofluorescence

Frozen tissue sections (4–6 µm) were immersed in ice-cold acetone for 10 min, followed by rehydration by immersion in PBS at room temperature. Rehydrated tissue slides were then blocked by immersion in 1% BSA in PBS for one hour. After blocking, slides were washed with blocking buffer, and a PAP pen was used to create a liquid blocking-barrier around the tissue. Next, the blocking buffer diluted anti-CD11b FITC antibody (diluted to manufacturer’s recommendation) was added gently to the enclosed tissue. Slides were then incubated for one hour in the dark in a humified chamber. After incubation, slides were washed in PBS, then mounted using Prolong Diamond Antifade Mountant with DAPI and covered with a 22 × 22 mm #1.5 coverslip. Coverslip corners were fixed using clear nail polish, then slides were allowed to dry overnight in the dark at room temperature. After 24 h, slides were stored at 4 °C, protected from light. Confocal images were acquired with a 63×/1.4 NA oil objective in sequential scanning mode using a Leica TCS SP8 confocal microscope.

### 2.14. Statistical Analysis

Experimental groups were compared using parametric, unpaired, equal variance student’s *t* Tests. Graphs were created using Graphpad Prism Version 10.0.0.

## 3. Results

### 3.1. MSN Design and Characterization

**Figure 1:**
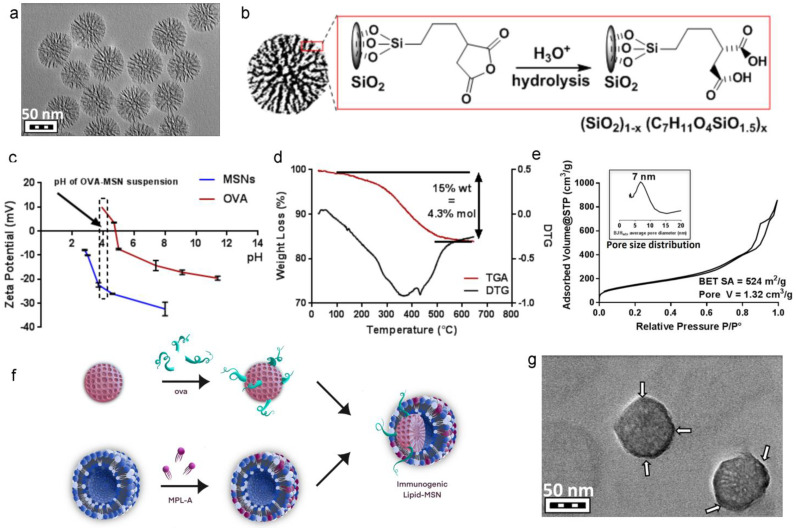
Synthesis of immunogenic lipid-coated mesoporous silica nanoparticles (ILM). (**a**) TEM image of MSNs. (**b**) Schematic showing MSN-COOH fabrication using established sol-gel methods, with hydrolysis of the grafted succinic anhydride yielding carboxylic acid groups. (**c**) pH dependence of zeta potential for free OVA and MSNs. The dotted line and arrow indicate OVA-loaded MSN. (**d**) TGA analysis of MSN-COOH. (**e**) N2 sorption isotherm with the inset indicating the BJH pore size (adsorption branch) distribution. (**f**) Schematic of MSN OVA loading and MPL-bearing liposome fusion. (**g**) TEM micrograph showing phosphotungstic acid-stained MSN coated by lipids (dark lipid halos are indicated by white arrows).

Dendritic MSNs with a pores size ~7 nm were made using established sol gel process using a biphase reaction that allows for fine-tuning of pore size and matrix density. TEM imaging shows monosized particles with a dendritic structure and 80 nm diameter (Figure 1a). In order to optimize ovalbumin loading into MSNs, both bare and carboxylic acid functionalized MSNs were made by co-condensing triethoxypropylsuccinic anhydride (5% mol compared to TEOS), which forms double carboxylic acid upon acid-treatment (Figure 1b). MSN-COOH outperformed bare MSN in stability upon protein loading and lipid fusion (*vide infra*, Figure S1) and hence was used for in vitro and in vivo studies. Zeta potential evolution of protein and MSN-COOH in function of pH was recorded (Figure 1c). It is noteworthy that ovalbumin acidifies the distilled water (1 mg/mL) to pH = 4.0, and importantly, the electrostatic interaction between the protein and the MSN is optimal at this pH (+10 mV and −25 mV, respectively), which is key for a successful loading (Figure 1c). The TGA curve in Figure 1d shows ~15% weight loss corresponding to the propyl di-carboxylic acid fragment (considering negligible continuation of silica condensation). This corresponds to ~4.3% mol (~80% co-condensation efficiency of the silane). Nitrogen sorption isotherm and porosity data are shown in Figure 1e, indicating a BET surface area ~524 m^2^/g and total pore volume of 1.32 cm^3^/g, where the average pore size diameter of 7 nm (BJH model) outlines the capacity of these MSN-COOH to accommodate OVA and potentially other cargo molecules in the future.

### 3.2. Optimization of Lipid Composition

**Figure 2:**
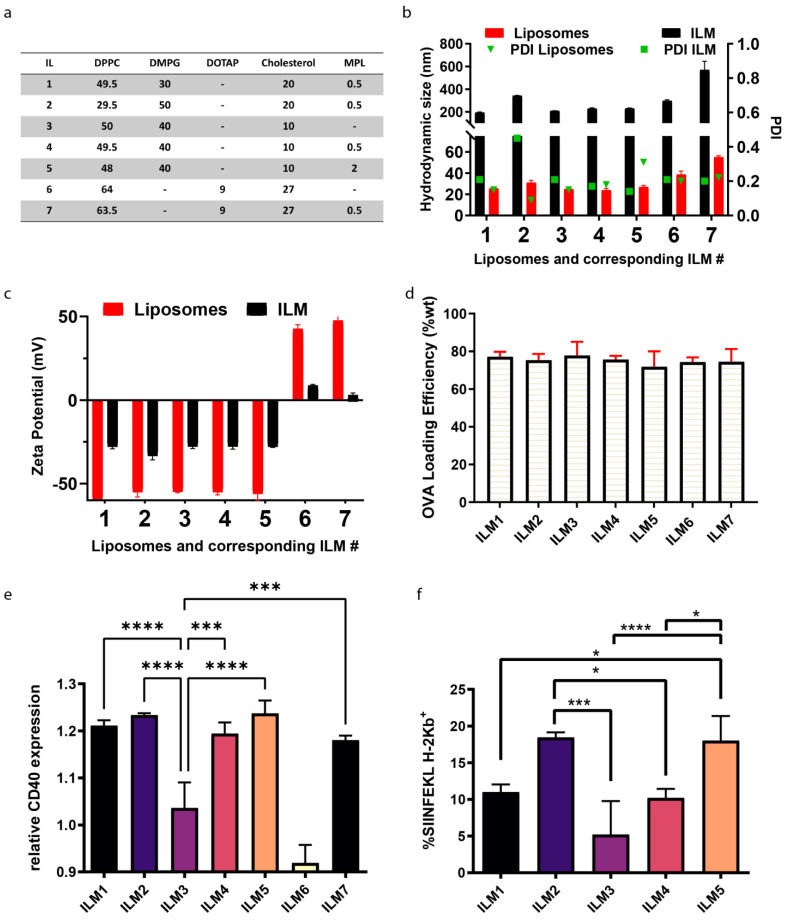
Optimization of nanoparticle composition for size, polydispersity (PDI), surface potential, and DC activation. (**a**) Lipid formulations (mol%). (**b**) Hydrodynamic size and PDI of free liposome (IL) or immunogenic lipid coated mesoporous silica (ILM) nanoparticles evaluated using dynamic light scattering. (**c**) IL or ILM surface potential for each formulation. (**d**) OVA loading based on lipid formulation. (**e**) CD40 expression by DC, following 72 h incubation with cationic or anionic ILMs. (**f**) MHC I peptide presentation (SIINFEKL-H-2Kb) by DC, following 72 h incubation with anionic ILM formulations. * *p* < 0.05, *** *p* < 0.001, **** *p* < 0.0001.

A schematic showing assembly of ILMs is presented in Figure 1f. Seven immunogenic lipid (IL) formulations were evaluated to find an optimal lipid composition that responds to four important criteria applicable to ILMs: (1) Colloidally stable and homogeneous, (2) optimal antigen presenting cell activation, (3) optimal antigen (ovalbumin) presentation and processing by antigen presenting cell, and (4) preferential uptake by myeloid cells which ideally requires the absence of stealth-promoting entities, such as PEG. All seven immunogenic liposomes (ILs) included DPPC as the skeleton lipid, along with cholesterol and anionic DMPG or cationic DOTAP before the incorporation of MPL-A at moderate dosage (0.2–2% mol) to endow the liposomes with an immunogenic character (immunogenic liposomes ILs). The homogeneity of ILs was evaluated by their hydrodynamic diameter and the corresponding polydispersity indices as well as the zeta potential. In parallel, ovalbumin solution in water (1 mg/mL) was added to the MSN pellet, and the final OVA-loaded MSN was designated OVA@MSNs. This step was followed by the introduction of the IL suspension to coat the OVA@MSN to yield ILMs. A negatively stained TEM micrograph shows a successful lipid coating on MSN (indicated by both change of contrast and masking of the morphological features of MSN) is presented in Figure 1g. The dark halo (indicated with arrows) depicts the lipid shell of the ILM. All ILM formulations evaluated are presented in Figure 2a. ILM hydrodynamic size and polydispersity (PDI) (Figure 2b), and zeta potential (Figure 2c) were assessed along with corresponding ILs. Quantitation of ovalbumin loading was similar for all formulations (Figure 2d).

### 3.3. DC Activation and Antigen Processing by ILM Formulation

DC activation by anionic (ILMs 1–5) and cationic (ILMs 6–7) ILMs, using CD40 surface expression as a metric, showed a high DC activation by all ILM except those lacking MPL-A (ILM3 and ILM6) (Figure 2e). SIINFEKL peptide presentation by major histocompatibility complex I (MHC I; H-2Kb) was superior using ILM2 and ILM5 (Figure 2f). Based on the superior polydispersity (near 0.2) and smaller colloidal size (200 nm), ILM5 (compared to ILM2) was chosen as the negative ILM for further in vitro and in vivo studies (hereafter referred to as anionic ILM or ILM^−^). Cationic ILM or ILM^+^ hereafter is used to indicate ILM7, the cationic formulation containing MPL-A. ILM- hydrodynamic size evolution during suspension in PBS or serum was evaluated and is presented in Figure S2. When suspended in serum at physiological temperature, ILM- are stable and maintain their size (over a 50 h time course). However, when immersed in PBS, charge interactions needed for nanoparticle stability are masked, altering ILMs size dramatically.

### 3.4. Cell Type-Dependent Internalization of Anionic ILM

**Figure 3:**
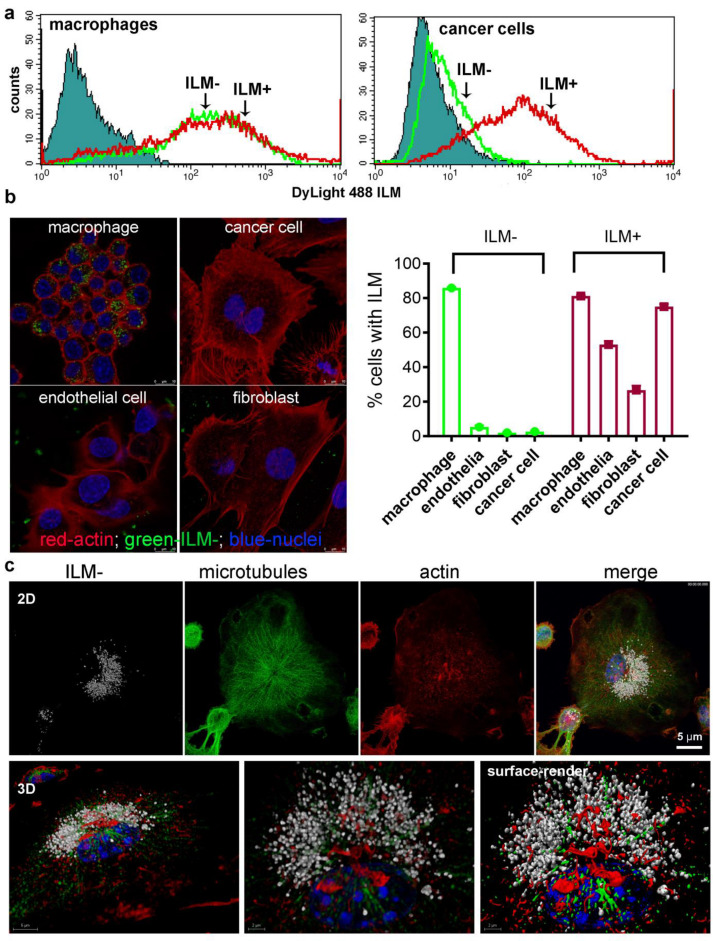
Non-myeloid cells fail to internalize ILM^−^ in the presence of 20% serum. (**a**) Flow cytometry analysis of macrophage or cancer cell uptake of fluorescent ILM^−^ or ILM^+^ after 24 h in media containing 20% serum. (**b**) Qualitative (confocal) and quantitative (flow cytometry) analysis of macrophage, fibroblast, endothelial, or cancer cell ILM uptake after 24 h. (**c**) Representative 2D and 3D images of a dendritic cell after 24 h incubation with ILM^−^.

Quantification of fluorescent ILM internalization was performed in the presence of 20% serum to reflect physiological uptake in the presence of serum proteins. RAW macrophages displayed equally high uptake of cationic and anionic ILM (Figure 3a). However, while non-myeloid cells (endothelial, fibroblasts, and cancer cells) were able to internalize cationic ILM, uptake of anionic ILM was negligible. Cell type dependent uptake of anionic ILM is further shown in fluorescent confocal images in Figure 3b. Consistent with macrophages, DC abundantly internalized anionic ILM. Indicative of endosomal trafficking, internalized ILM displaying perinuclear localization Figure 3c.

### 3.5. Lysosomal Tubulation and Streaming in DC following ILM Activation

**Figure 4:**
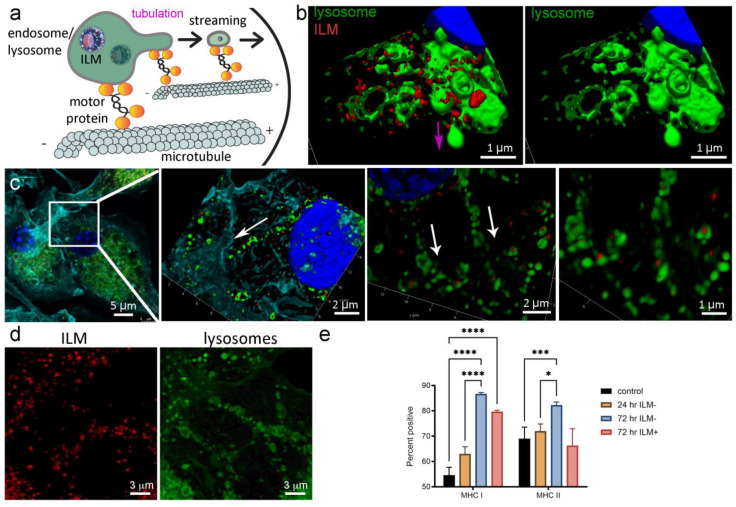
ILM stimulates endosomal tubulation and streaming in dendritic cells leading to enhanced MHC surface expression. (**a**) Schematic depicting endosome/lysosome tubulation and streaming. (**b**) 3D surface-rendered confocal micrographs showing lysosome tubulation originating from the nucleus and moving towards the cell surface (purple arrow) 24 h following ILM treatment (green, lysotracker; red DyLight™ 594 ILM). (**c**,**d**) 3D (**c**) and 2D (**d**), single fluorophore confocal micrographs showing lysosomes (green) containing ILM^−^ (red) streaming from the perinuclear region of the dendritic cell (white arrows). (**e**) Flow cytometry quantification of DC surface MHC I and MHC II expression 24 or 72 h post treatment with ILM^−^ or ILM^+^. * *p* < 0.05, *** *p* < 0.001, **** *p* < 0.0001.

Fused lysosomes and phagosomes are the major cellular compartment generated to digest microbes, with lysosomes being the major compartment that sends antigen complexed with MHC to the plasma membrane in macrophages and DCs, respectively [16]. LPS activation of macrophages and DCs stimulates numerous phenotypic changes, including the conversion of punctate lysosomes into dynamic tubular lysosomes that, with the aid of microtubules, form a scaffold to stretch lysosomes (Figure 4a) [16,17]. Tubular lysosomes have been indicated to have a role in antigen presentation [18]. Here, we show that the treatment of DC with MPL containing LM (i.e., ILM) stimulates lysosomal tubulation (Figure 4b) and streaming of vesicles (Figure 4c,d) towards the plasma membrane. Furthermore, surface MHC I was elevated on DC following treatment with anionic or cationic ILM (1.6- and 1.5-fold, respectively, compared to no treatment control cells). In addition, MHC II was elevated in response to anionic ILM (1.2-fold compared to no treatment control cells) (Figure 4e). This is consistent with TLR-mediated fusion of endo-/lyso-somes with Rab11a MHC storage vesicles, which leads to elevated surface MHC and antigen presentation [19,20].

### 3.6. ILM Biodistribution in a Mouse Model of Disseminated Ovarian Cancer

**Figure 5:**
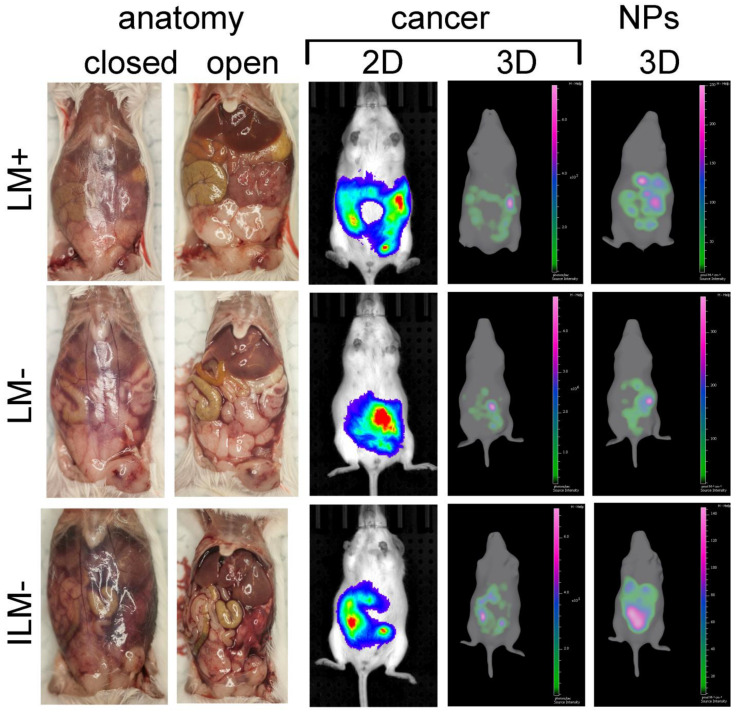
Gross anatomy and imaging of mice post nanoparticle administration. FVB female mice with disseminated peritoneal BR5-Akt-Luc ovarian cancer were injected intraperitoneally with DyLight™ 647 LM^+^, LM^−^ (no MPL), or ILM^−^. Photographs, bioluminescent, and fluorescent images of mice were acquired using the IVIS Spectrum 24 h later.

**Figure 6:**
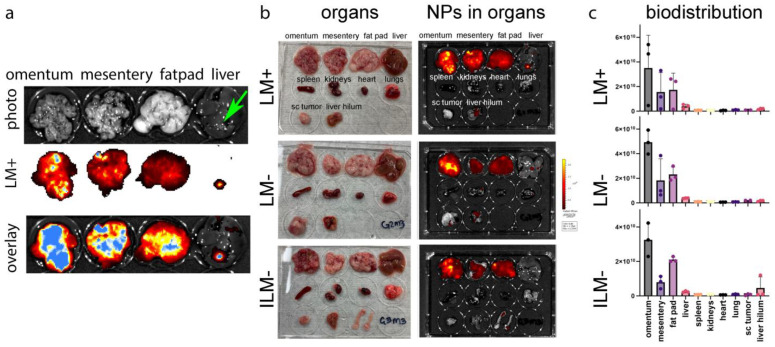
Intraperitoneal administration of ILM leads to selective accumulation in tumor-burdened tissues. FVB mice with disseminated peritoneal BR5-Akt-Luc ovarian cancer (Day 19) were injected intraperitoneally with DyLight™ 647 LM^+^, LM^−^ (no MPL), or ILM^−^. (**a**) Independent and merged photograph and fluorescent images of adipose tissues and liver. Green arrow indicates tumor on liver surface. (**b**) Photographs and fluorescent images of harvested tissues for each nanoparticle (NP) formulation. (**c**) graphs showing tumor accumulation by tissue type for each NP formulation.

Female FVB mice with disseminated (Day 19) BR5-akt-luc ovarian cancer (*n* = 3/group) were administered fluorescent ILM^−^ or LM (the latter lacking MPL-A; anionic or cationic) via intraperitoneal injection. Gross anatomy (with an open or closed peritoneal cavity) is shown for a representative mouse from each group in Figure 5. Bioluminescent and fluorescent images of cancer and nanoparticles, respectively, in live mice 24 h post nanoparticle injection are also presented, showing localization of both entities in the peritoneal cavity. Tissues from all mice were harvested and nanoparticle biodistribution was evaluated based on fluorescent signals. Figure 6a presents independent or merged photographs and fluorescent images of tumor-burdened adipose tissues from one mouse (enlarged to visualize the abundance of tumor nodules). Tissues from a representative mouse from each group are shown in Figure 6b, with all mouse tissues displayed in Figure S3. Nanoparticle biodistribution per organ is shown graphically in Figure 6c. Moreover, 74.5% ± 10.4 of all nanoparticles, regardless of charge or the presence of MPL-A, accumulated in tumor-burdened omentum, mesentery, and fatpads.

### 3.7. In Vivo Cellular Uptake of ILM

**Figure 7:**
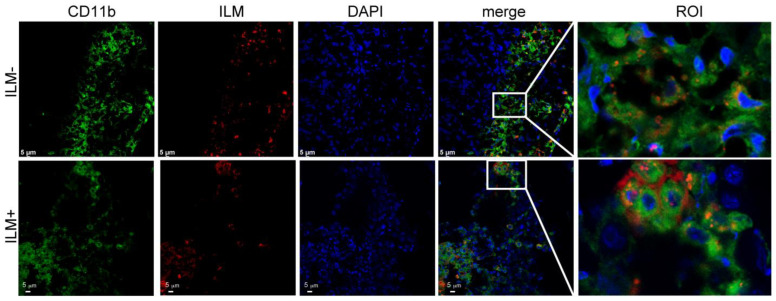
Cationic and anionic ILM are located within or near myeloid cells in omental tissues. Female FVB mice challenged intraperitoneally with BR5-Akt ovarian cancer cells (Day 19) were injected intraperitoneally with DyLight™ 647-labeled ILM. Omentum from representative mice were frozen in O.C.T, sectioned, fixed in acetone, and labeled with anti-CD11b fluorescent antibody 24 h later. Images were acquired with a Leica TCS SP8 confocal microscope.

Fluorescent imaging of omental tumor tissue from ILM-treated mice shows an accumulation of anionic and cationic ILM in myeloid (CD11b^+^)-rich areas 24 h after intraperitoneal administration (Figure 7). Merged confocal micrographs support in vivo internalization of anionic and cationic ILM by myeloid cells.

## 4. Discussion

For this study, a variety of ILM formulations were engineered to create nanoparticles capable of targeting, activating, and delivering antigen(s) to antigen presenting cells. Anionic ILMs were selectively internalized by myeloid cells in vitro compared to cationic ILMs, with the latter nonspecifically internalized by myeloid and non-myeloid cells (cancer, endothelial, and fibroblast). This phenomenon only occurs in the presence of serum based on differential opsonization of particles based on surface charge [21]. In addition to the specific uptake by myeloid cells, anionic ILMs stimulated a greater increase in surface MHC I and II expression on DC compared to cationic ILMs.

In 2018, Dogra et al. demonstrated that intraperitoneal delivery of 40–100 nm MSN in rats resulted in lymphatic transfer of MSN to the systemic circulation, leading to distribution across all organs initially, and then accumulating in filtering (sink-like) organs 24 h post administration [22]. Accumulation in the liver and spleen were similar to that following intravenous delivery. Haber et al. showed that intravenous injection of 500 nm silica nanoparticles resulted in a lack of accumulation in the peritoneal cavity of athymic nude mice injected intraperitoneally with human ovarian cancer cells. In contrast, intraperitoneal injection resulted in exclusive association of silica nanoparticles with tumor-burdened tissues with maximum accumulation at 4 days post injection [23]. Interestingly, in mice lacking tumors, no fluorescent silica particles were detected 4 days post intraperitoneal injection.

In this study, intraperitoneal administration of ILM in mice with disseminated ovarian cancer resulted in an almost exclusive localization of ILM within tumor-burdened tissues, regardless of particle charge and the presence of MPL-A. Haber et al. also showed that intraperitoneally delivered anionic silica, poly(lactic-co-glycolic acid), and polystyrene nanoparticles greater than 100 nm selectively accumulate in tumor-associated macrophages (TAMs) [23]. More than 80% of silica particles were located within TAMs. Silica nanoparticles smaller the 50 nm displayed minimal tumor targeting. In contrast to our findings, Haber et al. showed that cationic nanoparticles do not target TAMs. Major differences in the Haber study and the current work include the use of athymic nude mice, human cancer cells, lack of evaluation of 100 nm particles, and the lack of liposome coat. Both studies examined the biodistribution of nanoparticles near 20 days post intraperitoneal tumor challenge.

The omentum, largely composed of adipose tissue, is the primary site of attachment for initial disseminated ovarian cancer cells [24]. In both humans and mice, ovarian cancer cells bind selectively to immune aggregates within the omentum, known as milky spots. After adherence, the number of cancer cells in milky spots declines, while that in neighboring fatty tissues increases [25]. Secondary sites of dissemination include adipose tissues of the mesentery [26], which also contain fat-associated lymphoid clusters [27]. Many epithelial tumors metastasize to adipose tissue, with the latter developing cancer-associated adipocytes that provide a lipid-rich environment and paracrine signals that favor tumor growth [28,29]. Paradoxically, macrophages and other immune cells accumulate in milky spots. Here, we show that both anionic and cationic ILM associate with CD11b^+^ cells in the omentum 24 h post administration, consistent with myeloid uptake in the peritoneal fluid and trafficking of particles to secondary lymphoid structures in adipose tissues. Surprisingly, the presence of MPL-A within the lipid coat of the ILM did not alter in vivo biodistribution based on the abundant myeloid uptake of all particle variants.

As ovarian cancer progresses, the tumor microenvironment becomes increasingly immune suppressed [12]. TLR agonists have been shown to be effective at switching myeloid cells from a pro-tumorigenic and immunosuppressive (M2) phenotype to an anti-tumorigenic, immunostimulatory (M1) phenotype [30]. Tumor-specific accumulation of ILM and selective uptake by myeloid cells indicates that the ILM platform may be a valuable therapeutic tool for facilitating cancer-directed immune responses.

## 5. Conclusions

ILMs co-localize with omental immune cells (also known as milky spots) in female FVB mice 19 days post challenge with syngeneic BR5-akt-luc ovarian cancer cells, providing a vehicle with the potential to alter the tumor-suppressed microenvironment. Future studies will explore the therapeutic efficacy of ILMs loaded with tumor antigens and TLR agonists in mouse models of peritoneal cancer.

## Data Availability

All datasets generated and analyzed during the current study are available from the corresponding author upon reasonable request.

## Supplementary Materials

The following supporting information can be downloaded at: https://www.mdpi.com/article/10.3390/cancers15184626/s1, Figure S1: MSN-COOH form stable lipid-coated particles. (a) Colloidal size comparison of ILM made using bare MSN vs MSN-COOH particles. Based on superior size stability for ILM created with MSN-COOH, this core was used to create all ILMs studied. (b) Size, polydispersity index, and zeta potential measurements of ILs and their corresponding ILMs (made using MSN-COOH; numbers represent formulations presented in Figure 2a). Figure S2: Serum maintains ILM stability. DLS measurements of hydrodynamic size evolution of ILM- during incubation in PBS or serum (FBS) at physiological temperature over 50 h. Figure S3: Nanoparticles accumulate in tumor-burdened peritoneal tissues 24 h post intraperitoneal administration. Fluorescent LM and ILM- were administered by intraperitoneal injection to female FVB mice 19 days post BR5-akt tumor challenge. Images of harvested peritoneal organs were acquired 24 post nanoparticle injection with omentum, mesentery and fatpad shown in the first 3 wells of each organ presentation.
