# Supplementary material for: Specific Tumor Localization of Immunogenic Lipid-Coated Mesoporous Silica Nanoparticles following Intraperitoneal Administration in a Mouse Model of Serous Epithelial Ovarian Cancer
Source: Cancers (Basel). 2023 Sep 19;15(18):4626. doi: 10.3390/cancers15184626 (PMC10526288; doi:10.3390/cancers15184626)
Supplement: Supplementary file 1 [file cancers-15-04626-s001.zip › cancers-2570311-supplementary.pdf]

Supplemental Data

Specific tumor localization of immunogenic lipid-coated mesoporous silica nanoparticles following intraperitoneal administration in a mouse model of serous epithelial ovarian cancer

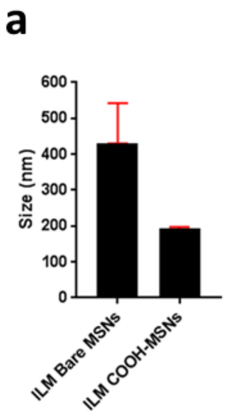

b

	Immunogenic Liposomes (IL)			Immunogenic Lipid-Coated MSNs (ILM)		
	Size (std dev) (nm)	PDI (± std dev)	Zeta Potential (std dev) (mV)	Size (std dev) (nm)	PDI (std dev)	Zeta Potential (std dev) (mV)
1	25.6 (0.7)	0.15 (0.02)	-61.6 (0.9)	194.1 (4.3)	0.21 (0.01)	-27.8 (1.33)
2	31.0 (2.2)	0.09 (0.01)	-55.1 (2.93)	342.4 (3.4)	0.45 (0.03)	-33.3 (2.41)
3	24.9 (1.6)	0.15 (0.01)	-54.8 (0.7)	208.8 (1.5)	0.21 (0.01)	-27.8 (1.14)
4	23.8 (1.8)	0.18 (0.02)	-55.1 (1.67)	229.8 (6.4)	0.17 (0.03)	-27.8 (1.56)
5	27.0 (1.4)	0.31 (0.01)	-56.1 (5.28)	230.6 (3.8)	0.14 (0.02)	-27.9 (0.49)
6	38.7 (3.3)	0.20 (0.01)	42.6 (2.52)	297.8 (7.8)	0.21 (0.05)	8.8 (0.65)
7	55.1 (1.3)	0.22 (0.01)	47.6 (3.24)	569.3 (76.1)	0.20 (0.17)	3.0 (1.27)

Fig. S1. MSN-COOH form stable lipid-coated particles. a) Colloidal size comparison of ILM made using bare MSN vs MSN-COOH particles. Based on superior size stability for ILM created with MSN-COOH, this core was used to create all ILMs studied. b) Size, polydispersity index, and zeta potential measurements of ILs and their corresponding ILMs (made using MSN-COOH; numbers represent formulations presented in Fig. 2a).

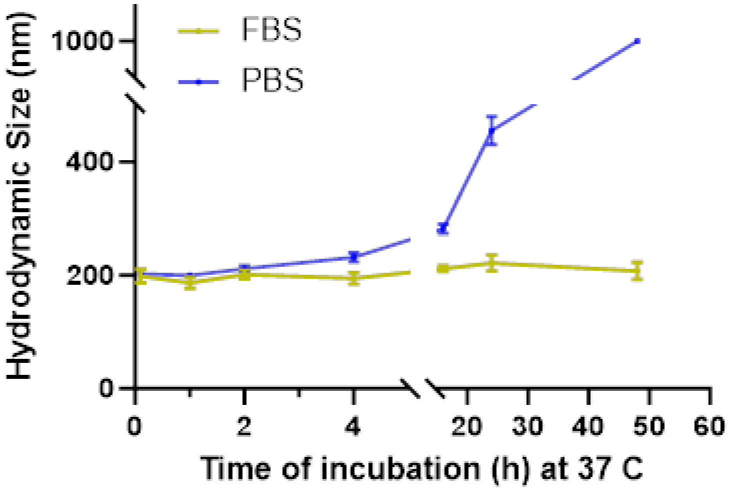

Fig. S2. Serum maintains ILM stability. DLS measurements of hydrodynamic size evolution of ILM^- during incubation in PBS or serum (FBS) at physiological temperature over 50 hours.

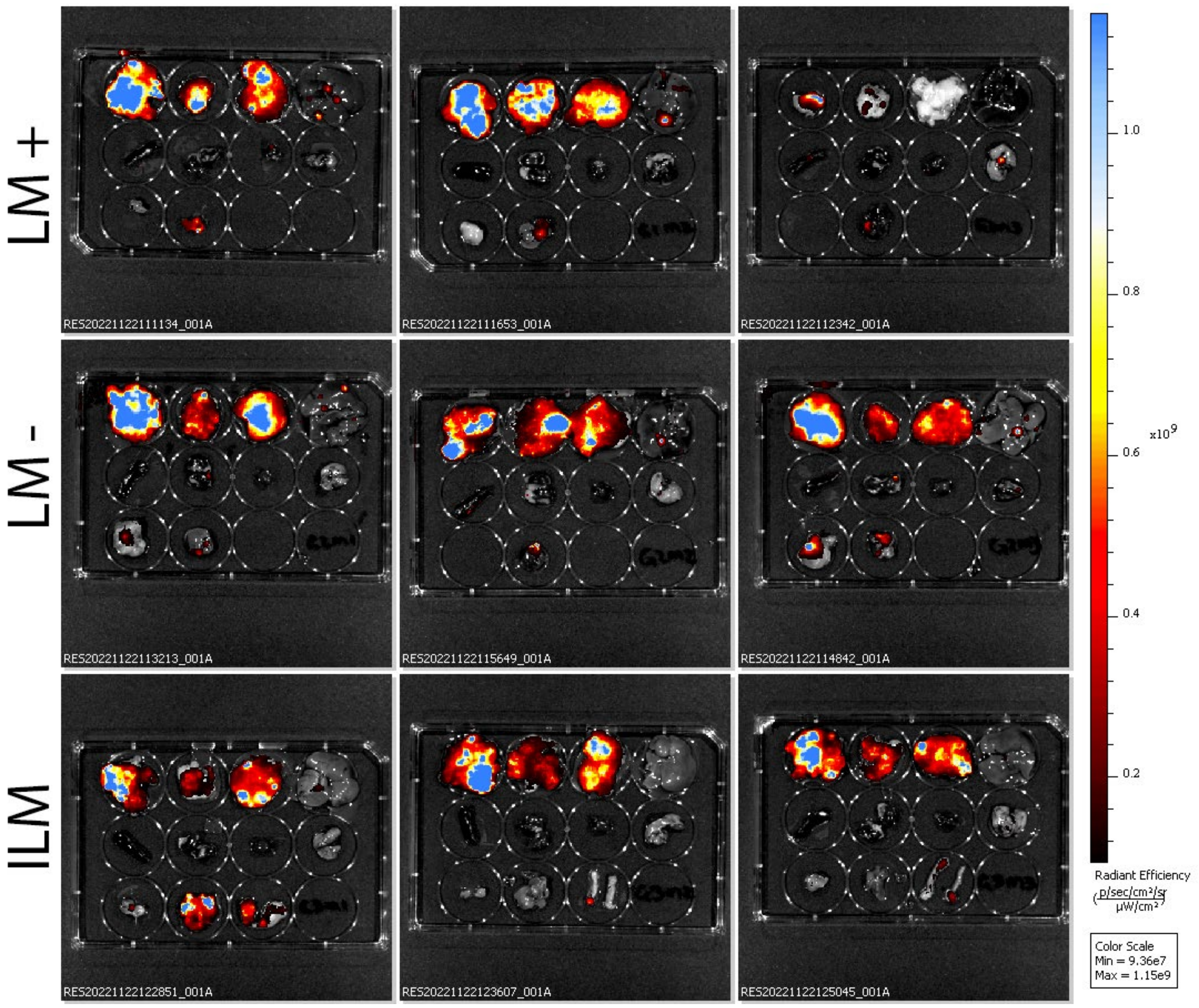

Fig. S3. Nanoparticles accumulate in tumor-burdened peritoneal tissues 24 hours post intraperitoneal administration. Fluorescent LM and ILM- were administered by intraperitoneal injection to female FVB mice 19 days post BR5-akt tumor challenge. Images of harvested peritoneal organs were acquired 24 post nanoparticle injection with omentum, mesentery and fatpad shown in the first 3 wells of each organ presentation.
